# Stable and Fast Planar Jumping Control Design for a Compliant One-Legged Robot

**DOI:** 10.3390/mi13081261

**Published:** 2022-08-05

**Authors:** Guifu Luo, Ruilong Du, Sumian Song, Haihui Yuan, Zhiyong Huang, Hua Zhou, Jason Gu

**Affiliations:** 1 State Key Laboratory of Fluid Power and Mechatronic Systems, Zhejiang University, Hangzhou 310027, China; 2 Intelligent Robot Research Center, Zhejiang Lab, Hangzhou 311100, China; 3 Department of Electrical Engineering, Dalhousie University, Halifax, NS B3J 1Z1, Canada

**Keywords:** planar jumping, compliant one-legged robots, control strategy, stable and fast locomotion, robustness, uneven terrain

## Abstract

Compliant bipedal robots demonstrate a potential for impact resistance and high energy efficiency through the introduction of compliant elements. However, it also adds to the difficulty of stable control of the robot. To motivate the control strategies of compliant bipedal robots, this work presents an improved control strategy for the stable and fast planar jumping of a compliant one-legged robot designed by the authors, which utilizes the concept of the virtual pendulum. The robot was modeled as an extended spring-loaded inverted pendulum (SLIP) model with non-negligible torso inertia, leg inertia, and leg damping. To enable the robot to jump forward stably, a foot placement method was adopted, where due to the asymmetric feature of the extended SLIP model, a variable time coefficient and an integral term with respect to the forward speed tracking error were introduced to the method to accurately track a given forward speed. An energy-based leg rest length regulation method was used to compensate for the energy dissipation due to leg damping, where an integral term, regarding jumping height tracking error, was introduced to accurately track a given jumping height. Numerical simulations were conducted to validate the effectiveness of the proposed control strategy. Results show that stable and fast jumping of compliant one-legged robots could be achieved, and the desired forward speed and jumping height could also be accurately tracked. In addition to that, using the proposed control strategy, the robust jumping performance of the robot could be observed in the presence of disturbances from state variables or uneven terrain.

## 1. Introduction

**Figure 1:**
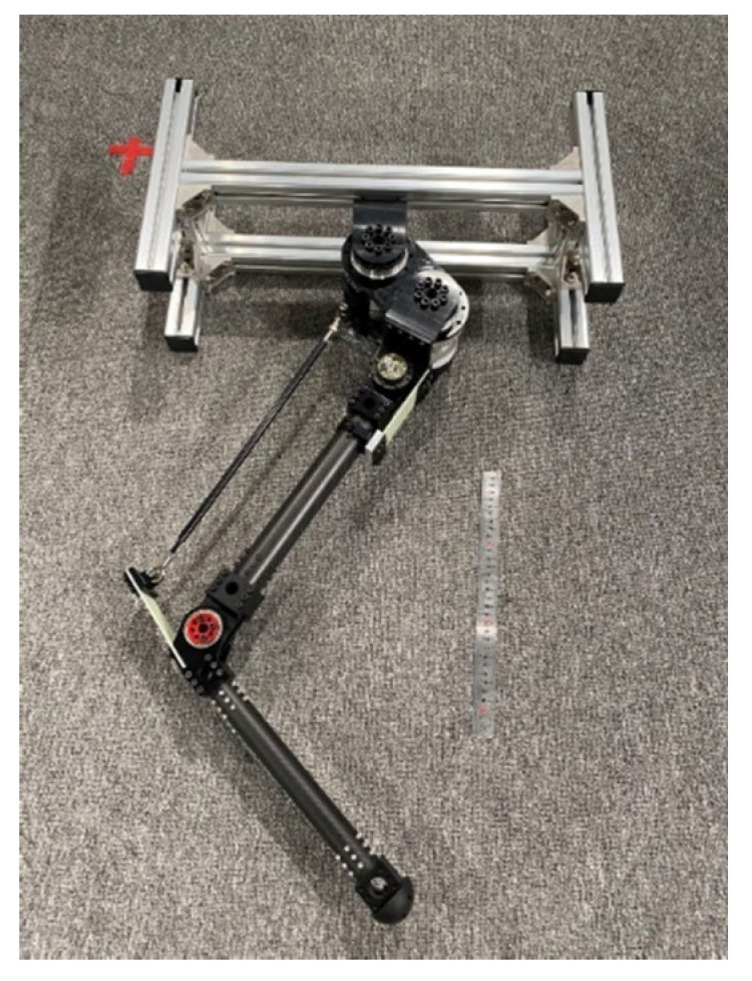
The compliant one-legged robot which was designed by ourselves.

Bipedal robots have shown strong application prospects due to their humanoid characteristics and flexibility of locomotion. In the past two decades, several bipedal robots were successfully developed, which could achieve high anthropomorphic stable locomotion. For instance, ASIMO [1], developed by Honda, could perform actions such as going up and down stairs, jumping, and kicking a ball; HRP-5P [2], developed by AIST, could cooperate with hands and feet to manipulate a gypsum board; Atlas [3], developed by Boston Dynamics, could perform a series of complex actions, such as parkour. Although robots have exhibited the capability of achieving complex locomotion, they face a huge challenge of high energy consumption [4]. By introducing passive compliance, bipedal robots exhibit the potential of impact resistance and high energy efficiency. For example, through introducing a passive compliant ankle, the cost of the electrical transport of DURUS is much lower than that of ASIMO and Atlas [5]; through introducing a passive compliant kinematics chain, the energy consumption of Cassie is closer to that of human [4]. By introducing passive compliance, the shock tolerance capacity of bipedal robots is also enhanced, and agiler locomotion can be achieved [6]. However, the passive compliant parts increase the degrees of freedom of robot dynamics, adding to the difficulty of stable control of the robots. This work aims to explore an improved control strategy for stable and fast planar jumping of a compliant one-legged robot designed by ourselves, as shown in Figure 1, thereby providing a reference for the control strategy of compliant bipedal robots.

Different modeling methods and control strategies could be found for the stabilization and fast locomotion of compliant legged robots, whether based on full-order models or simplified models. Regarding the full-order model, Westervelt et al. [7] proposed virtual constraints and the hybrid zero dynamics (HZD) method to address the gait generation problem for underactuated legged robots. Based on these concepts, related research was further developed and successfully applied to real robots. Da et al. [8] elevated the 2D underactuated bipedal walking gait to 3D by utilizing virtual constraints and the HZD method, and then, they combined it with the gait library method to achieve speed switching within a certain range on ATRIAS. Reher et al. [9] implemented compliant walking on Cassie, for variable walking speed cases, by combining a Lyapunov function-based real-time controller, HZD method, and the gait library method. The main advantages of the full-order model-based control strategies are high control accuracy and mathematically provable stability; however, they need a certain amount of accurate information about the dynamic system and are computationally intensive [10,11]. Thus, it is of great significance to explore simplified modeling and related control strategies for stable and fast locomotion of compliant robots. 

Inspired by human and animal jumping and running, Blickhan et al. [12,13] proposed the spring-loaded inverted pendulum (SLIP) model as a template model to describe the locomotion of legged animals and robots. It assumes that the total mass of the robot is concentrated at a single point in the hip joint and that there are one or two massless linear spring-like legs attached under the mass point. It allows for the approximation of the trajectory of the center of mass (CoM) and the ground reaction force (GRF), of biological locomotion with different leg configurations, and inspires stable control methods for legged robots. Raibert [14] introduced the well-known three-part controller and successfully applied it to Raibet’s Hopper, and this controller greatly inspired the stable control strategies for the SLIP model. Rezazadeh et al. [15] modeled ATRIAS as a two-leg SLIP model, used periodic time-based trajectories for the leg rest length regulation, used a discrete PID foot placement method for horizontal speed control, and implemented the robust walking and running of ATRIAS in unstructured environments. Xiong et al. introduced an actuated SLIP model for jumping and walking gait generation, which was successfully implemented on the legged robot Cassie [16,17]. Some other SLIP model-based robot control strategies can be seen in [18,19,20,21]. However, the SLIP model neglected information about the torso and legs, such as inertia and damping, which makes it difficult to stabilize the torso and obtain accurate locomotion.

Noticing this, Maus et al. [22] introduced an extended SLIP model with a rigid torso and spring legs, and they proposed a posture control method based on the virtual pendulum concept for the stable control of the upright walking and running of legged robots. Then, from the perspective of bionics, a virtual pivot concept was further proposed to better understand and make use of the principle of locomotion in humans and animals [23]. The extended SLIP model and the virtual pendulum concept were further developed, by other researchers, for robust control of legged robots hopping and walking [24,25]. However, the abovementioned control method could not adaptively track the desired forward speed and jumping height of the robot modeled by the extended SLIP model, and more details about the limitations of these controllers could be seen in Section 4. To the best knowledge of the authors, few works except the abovementioned could be found implementing stable and fast compliant one-legged robot jumping by combining the extended SLIP model and the concept of the virtual pendulum, which is of great significance to make full use of the bio-inspired control method for extending the abilities of compliant legged robots. 

The main contributions of this work are concluded as follows:

(1) This work implements the stable forward jumping of a one-legged robot with a non-negligible torso modeled by the extended SLIP, and the robot’s forward speed and jumping height can be adaptively tracked at the same time.
(2) An improved jumping control strategy, based on the three-part control scheme and the concept of the virtual pendulum, was proposed for achieving the stable and fast jumping mentioned above. To track the desired forward speed adaptively, we introduced the integral term and the variable time coefficient to improve the speed control method for speed regulation; to track the desired jumping height adaptively and accurately, we improved the energy regulation method for the jumping height control of the extended SLIP model by introducing a jumping height error-based energy updating method.
(3) Comparative simulations were conducted to demonstrate the control accuracy for jumping speed and jumping height tracking. Additional simulations, regarding different desired forward speeds up to 6.3 km/h, jumping with state perturbations, and jumping on uneven terrain, were carried out to further demonstrate the performance of the proposed controller.

The rest of this work is organized as follows. Section 2 briefly describes the compliant one-legged robot designed by ourselves. Section 3 depicts the dynamic modeling of the robot’s jumping, taking the torso inertia, leg inertia, and leg damping into account. Section 4 introduces the design of the controller that enables the robot to jump stably. Afterward, Section 5 displays the simulation results, including control accuracy and stability analysis, as well as the robot’s stable and fast jumping performance. Lastly, Section 6 concludes this work and presents future work.

## 2. Brief Description of the Planar Compliant One-Legged Robot

**Figure 2:**
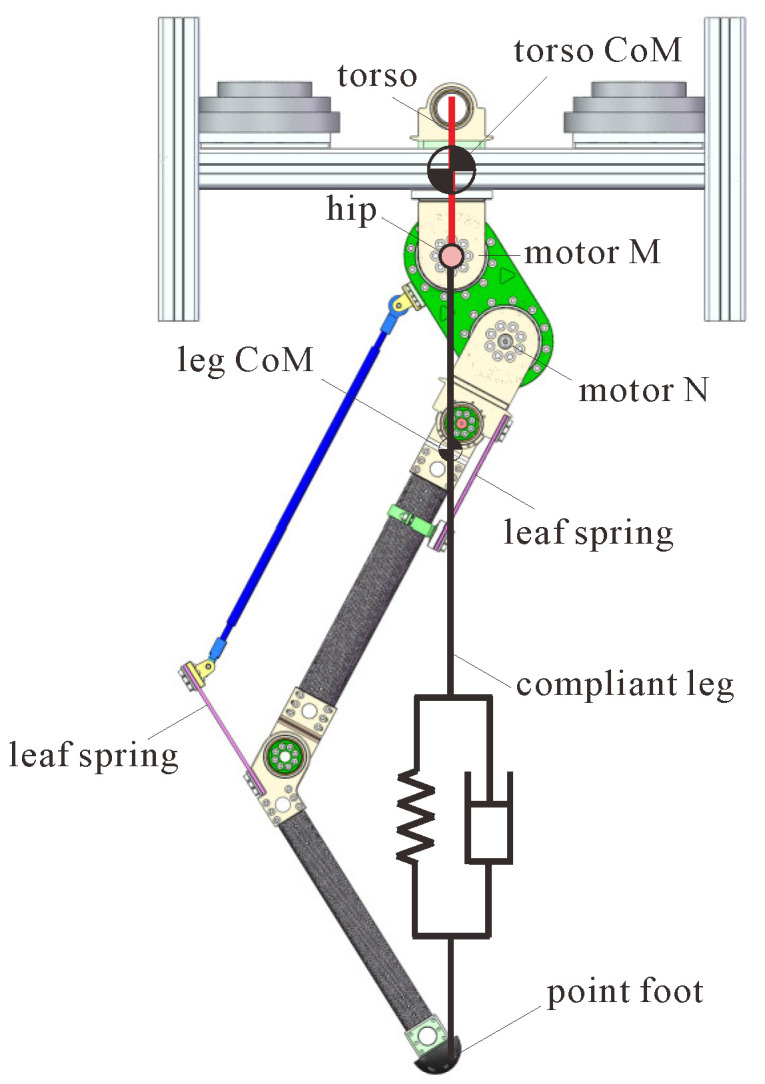
CAD model of our compliant one-legged robot and its related extended SLIP model.

Figure 2 presents the CAD model of the planar compliant one-legged robot in this work, whose leg dimension is based on human leg proportions. The robot mainly consists of two parts: a 12 kg torso, with its CoM offset from the hip, and a 6.5 kg leg that has two actuated motors connected in series, which are a thigh consisting of a compliant linkage mechanism using two leaf springs and a point-footed shank.

**Figure 3:**
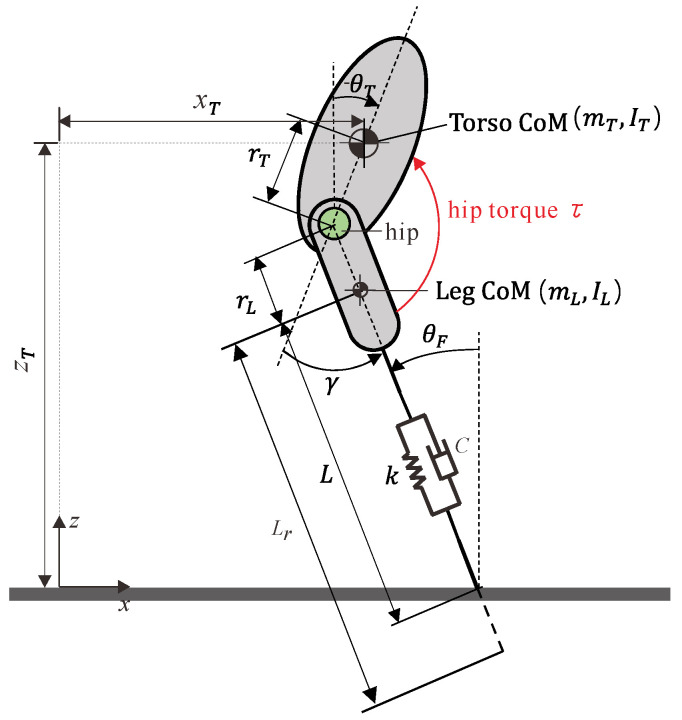
Extended SLIP model for our compliant one-legged robot.

The motors M and N are responsible for the swing angle and length adjustment of the rest length of the leg, respectively. The leg length can reach around 900 mm under full extension and 300 mm under full contraction, while the initial value was set as 700 mm in this work. By introducing a compliant six-bar linkage mechanism that includes two leaf springs in the thigh, the length of the leg will change when subjected to a force along the leg, just as with a spring, thus enabling the application of the extended SLIP model. To simplify the modeling of the leg, the leg was divided into two parts: a rigid part that refers to the part from the hip joint to the leg CoM, whose length would not change, and a spring part that refers to the part from the leg CoM to the pointed foot, whose length would change concerning ground reaction force. Table 1 displays some design parameters of the simplified robot model shown in Figure 3, and for more details on the robot design, one can refer to our previously published work [26,27].

## 3. Modeling of the Designed Robot

Figure 3 presents the extended SLIP model with a non-negligible torso and leg mass for the planar jumping of the compliant one-legged robot. As described above, the motor N, which is responsible for leg rest length adjustment, will inject or release energy for different jumping targets, such as a certain jumping height or a certain forward speed, while motor M will provide active hip torque τ for the swing angles between the leg and torso.

Regarding the definition of the world coordinate for the robot’s jumping, the *x*-direction, and the *z*-direction were defined as the forward and hopping directions, respectively. $x_{T},z_{T},\;\theta_{T}$ denote translational displacement in the *x*-direction and the *z*-direction and rotation angle concerning the *y*-direction of the torso, respectively; γ denotes the rotation angle of the leg from the axis along the torso to the leg, i.e., swing angle of the leg; $L_{r},\;L$ denote the leg rest length and leg length due to the spring-like deformation; $\theta_{F}$ denotes the foot angle and was named as the angle of attack at the moment the foot touches the ground.

The jumping dynamics of the robot could be divided into three successive processes: the flight phase in the air, the stance phase on the ground, and the transition process between these two phases. The dynamics modeling of these processes will be described, in detail, in the following subsections. It is worth noting that the former two phases can be described by continuous dynamics equations.

### 3.1. Continuous Dynamics

#### 3.1.1. Flight Phase

During the flight phase, the foot of the robot does not touch the ground. Therefore, the extended SLIP model for the flight phase features four degrees of freedom (DoFs), and here, we selected $q_{f}=\left(x_{T};z_{T};\;\theta_{T};\;\gamma \right)$ as the generalized variables. The dynamics equations for the flight phase can be expressed as:

(1) $$M_{f}\left(q_{f}\right){\ddot{q}}_{f}+C_{f}\left(q_{f},{\dot{q}}_{f}\right){\dot{q}}_{f}+G_{f}\left(q_{f}\right)=B_{f}u_{f}$$

where $q_{f}\in Q_{f}$ and $Q_{f}$ represents the configuration space of the flight phase dynamics, $M_{f}\in R^{4\times 4},\;C_{f}\in R^{4\times 4},\;G_{f}\in R^{4}$ represent the inertia matrix, Coriolis and centrifugal matrix, and gravity vector, respectively, $B_{f}=I_{4\times 4}$ represents the coefficient matrix of generalized forces, and $u_{f}=\left(0;0;0;\tau \right)$ represents the vector of generalized forces. 

#### 3.1.2. Stance Phase

During the stance phase, the foot of the robot is in contact with the ground, and under the assumption that it does not slip against the ground, the foot plays the role of a pivot joint. Due to the spring-like compliance property of the leg, the leg length L would change passively under the ground reaction force (GRF). Therefore, the extended SLIP model for the stance phase features three degrees of freedom (DoFs), and here, we selected $q_{s}=\left(\theta_{T};\;\gamma ;L\right)$ as the generalized variables. Thus, the dynamics equations for the stance phase can be expressed as:

(2) $$M_{s}\left(q_{s}\right){\ddot{q}}_{s}+C_{s}\left(q_{s},{\dot{q}}_{s}\right){\dot{q}}_{s}+G_{s}\left(q_{s}\right)=B_{s}u_{s}$$

where $q_{s}\in Q_{s}$ and $Q_{s}$ represents the configuration space of the stance phase dynamics, $M_{s}\in R^{3\times 3},\;C_{s}\in R^{3\times 3},\;G_{s}\in R^{3}$ represent the inertia matrix, Coriolis and centrifugal matrix, and gravity vector in stance phase, respectively, $B_{s}=I_{3\times 3}$ represents the coefficient matrix of generalized torques, $u_{s}=\left(0;\tau ;\;F_{s}\right)$ represents the vector of generalized torques, and $F_{s}=-k\left(L-L_{r}\right)-c\dot{L}$ represents the passive spring and damping force.

According to the two equations above, the state space expressions of the continuous dynamics for the extended SLIP model can be described as:

(3) $${\dot{x}}_{i}=\frac{d}{\mathrm{dt}}\left[\begin{array}{c} q_{i}\\ {\dot{q}}_{i} \end{array}\right]=\left[\begin{array}{c} {\dot{q}}_{i}\\ -M_{i}^{-1}\left(q_{i}\right)\left(C_{i}\left(q_{i},{\dot{q}}_{i}\right)+G_{i}\left(q_{i}\right)\right) \end{array}\right]+\left[\begin{array}{c} 0\\ M_{i}^{-1}\left(q_{i}\right)B_{i} \end{array}\right]u_{i}$$

where i={f,s} represents one of the different continuous phases mentioned above, $x_{i}=\left(q;\dot{q}\right)\in TQ_{i}$ represents the state vector, and $TQ_{i}$ represents the related state space.

### 3.2. Transit Maps

As seen in Equations (1) and (2), different DoFs and generalized variables could be observed between the flight phase and the stance phase, and therefore, transit maps between the state variables regarding different phases are needed. The transit maps consist of two sub-maps, the touchdown map, and the lift-off map.

#### 3.2.1. Touchdown Map

The touchdown map refers to the map in which the state variables in the flight phase are transformed into the state variables in the stance phase. The touchdown event occurs when the foot of the robot touches the ground, characterized by the displacements of the foot in the *x*-direction and *z*-direction reducing to zero. Two issues need to be addressed: the transient change of state variables due to the impact on the ground and the transformation of the different state variables regarding the two phases.

Regarding the impact with the ground, it is considered to occur at the moment of touchdown and to be a completely inelastic collision, which could be taken as an impulse that brought the velocity of the foot to zero instantaneously [7]. Moreover, due to our compliant leg design and that we only consider the mass above the spring-like leg, the impulse force along the leg will be zero, acting only in the direction perpendicular to the leg. To address the dynamic feature at the impact moment, adding the impact force δλ∈R into Equation (1), the equations for the constrained dynamics, in this process, can be expressed as:

(4) $$M_{f}\left(q_{f}\right){\ddot{q}}_{f}+C_{f}\left(q_{f},{\dot{q}}_{f}\right){\dot{q}}_{f}+G_{f}\left(q_{f}\right)+A^{T}\left(q_{f}\right)\delta \lambda =B_{f}u_{f}$$

where $A\in R^{4\times 1}$ represents the constraint matrix, and $\delta \lambda \in R^{1}$ represents the impulse force vector during impact. Integrating both sides of Equation (4), the touchdown map can be expressed as:

(5) $$M_{f}\left({\dot{q}}_{f}^{+}-{\dot{q}}_{f}^{-}\right)+A^{T}\lambda =0$$

where $\lambda \in R^{1}$ represents the intensity of the impulse force, and ${\dot{q}}_{f}^{-},\;{\dot{q}}_{f}^{+}$ represent the generalized velocities of the robot before and after the impact. The orientation of the leg is in parallel with the straight line passing through the hip joint and the contact point during impact, making the leg confined to a holonomic constraint expressed as follows:

(6) $$h=\tan\left(\theta_{F}\left(q_{f}\right)\right)-\frac{y_{hip}\left(q_{f}\right)-y_{con}}{x_{hip}\left(q_{f}\right)-x_{con}}=0$$

where $\left(x_{hip},\;y_{hip}\right)$ and $\left(x_{con},\;y_{con}\right)$ represent the coordinate of the hip joint and the contact point of the robot in the world coordinate.

The relative velocity constraint equation can be expressed as:

(7) $$\dot{h}\left(q_{f}\right)=\frac{\partial h}{\partial q_{f}}\;{\dot{q}}_{f}=A\left(q_{f}\right){\dot{q}}_{f}=0$$

Combining Equations (5) and (7), the impulse intensity and the generalized velocities after impact can be derived as:

(8) $$\lambda ={\left(AM_{f}^{-1}A^{T}\right)}^{-1}A{\dot{q}}_{f}^{-}$$

(9) $${\dot{q}}_{f}^{+}={\dot{q}}_{f}^{-}-M_{f}^{-1}A^{T}\lambda =\Delta_{imp}{\dot{q}}_{f}^{-}$$

Regarding the transformation of the different state variables between the flight phase and the stance phase, the following expression can be used:

(10) $$x_{s0}=\Delta_{f\to s}\left(x_{f}^{+}\right)$$

where $x_{s0}=\left(q_{s0};{\dot{q}}_{s0}\right)\in TQ_{s}$ denotes the initial states of the stance phase; $x_{f}^{+}=\left(q_{f}^{+};{\dot{q}}_{f}^{+}\right)\in TQ_{f}$, represents the flight states after impact; $\Delta_{f\to s}:TQ_{f}\to TQ_{s}$ represents the reset map from flight to stance.

#### 3.2.2. Lift-Off Map

The lift-off map refers to the map in which the state variables in the stance phase are transformed into those in the flight phase. The lift-off event occurs when the leg force $F_{s}$ tends to be less than 0 N. No impact would occur since the ground cannot impose pull force on the robot, and thus, we only need to address the reset map of state variables from the stance phase to the flight phase:

(11) $$x_{f0}=\Delta_{s\to f}\left(x_{s}^{-}\right)$$

where $x_{f0}=\left(q_{f0};{\dot{q}}_{f0}\right)\in TQ_{f}$ denotes the initial state variables in the flight phase; $x_{s}^{-}=\left(q_{s}^{-};{\dot{q}}_{s}^{-}\right)\in TQ_{s}$, denotes ending states of the stance phase; $\Delta_{s\to f}:TQ_{s}\to TQ_{f}$ denotes the reset map from flight to stance.

## 4. Control Design

### 4.1. Control Strategy

**Figure 4:**
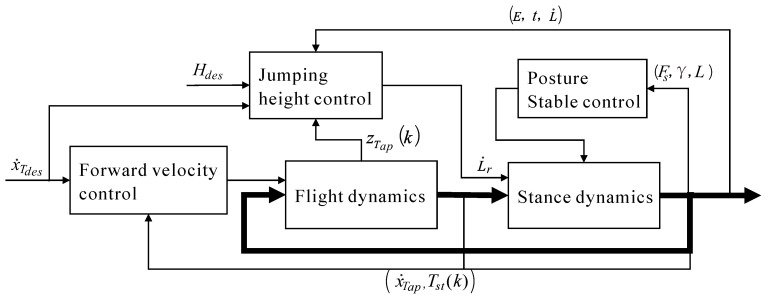
Block diagram of the control strategy.

Figure 4 presents the block diagram of the control strategy for the stable and fast planar jumping of the compliant one-legged robot. The flight dynamics and stance dynamics blocks represent the dynamics of the flight phase and stance phase of the jumping, respectively. The bold black lines between flight dynamics and stance dynamics represent all state information of the jumping of the extended SLIP model (e.g., the state variables $x_{s}$ and $x_{f}$, ground reaction force $F_{c}$, and system energy E). The state information with *k* means that it was observed at a certain discrete event during the *k*th jumping, such as $x_{T_{ap}}\left(k\right)$ represents the forward displacement of the torso CoM measured at the *k*th apex, and $T_{st}\left(k\right)$ represents the stance period of the *k*th jumping; while others are observed continuously, such as the system energy E,  the spring-like leg deformation rate $\dot{L}$, etc. The control strategy consists of three parts: posture stable control, jumping height control, and forward speed control. In the stance phase, posture stable control based on the virtual pendulum concept keeps the torso from tripping over, and jumping height control makes the legged robot track the desired jumping height accurately through energy injection or releasing. In the flight phase, forward speed control makes the robot track the desired forward speed ${\dot{x}}_{T_{des}}$. The thin lines and corresponding texts represent the input and output signals of three control parts. With this control scheme, stable and fast jumping of the compliant one-legged robot could be achieved. The details of the three control parts were presented in the following subsections.

#### 4.1.1. Posture Stable Control

**Figure 5:**
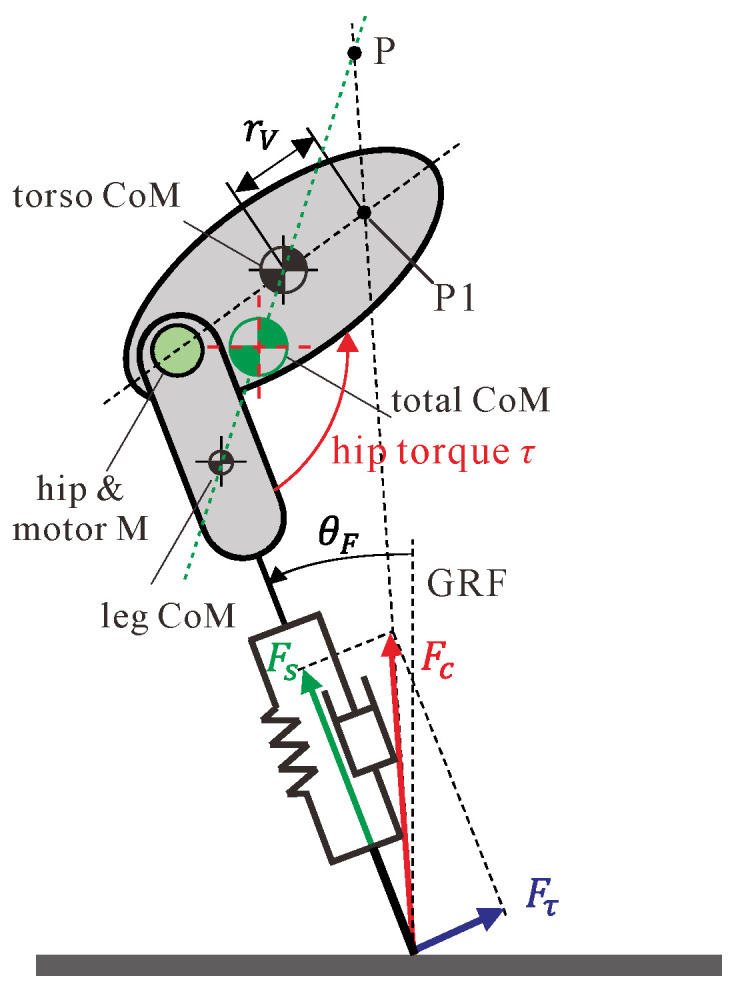
Schematic of the VPPC-FP control strategy.

Regarding the stance phase, as depicted in Figure 5, a simple and robust controller, called approximate virtual pendulum posture control with fixed point (approximate-VPPC-FP) [24], was adopted. In [23], this controller was validated by experimental results of biological legged locomotion systems, such as walking humans and running chickens, etc.

As shown, the ground reaction force (GRF) $F_{c}\;$is the combining force of two orthogonal directions: along the leg and perpendicular to the leg. The force $F_{s}$ along the leg is the passive force produced by the deformation of the leg spring and damping, and it is defined as $F_{s}=-k\left(L-L_{r}\right)-c\dot{L}$, while the force which is perpendicular to the leg $F_{\tau }$ is produced by the hip torque τ from motor M and influenced by the dynamics of the leg and torso. Via regulating the hip torque τ, it is expected that $F_{c}$ is directed to a point P located above the total CoM of the robot, which is called the virtual pivot point (VPP). When the point P is fixed to a constant distance above the total CoM, it can be seen that the total CoM rotates around the point P such as how a physical pendulum would, which is a naturally stable system. Thus, it is said that the point P and the total CoM form a virtual pendulum [24].

In this work, to simplify the control strategy, we leave the VPP located at the point P1, which is on the axis of the torso, at a distance of $r_{v}$ from the torso CoM, where $r_{v}$ is positive when the point is on the right and negative when on the left. It is reasonable for this simplification because the point P1 and the torso CoM also form a virtual pendulum, which can also stabilize the torso. The value of $r_{v}$ needs to be chosen carefully, and when the leg mass was neglected, $F_{\tau }$ arises entirely from the hip torque τ, according to the moment balance of the leg. Then, the approximate hip torque τ yields:

(12) $$\tau =-F_{\tau }=-F_{s}\left(r_{L}+L\right)\frac{\left(r_{T}+r_{v}\right)\sin\left(\gamma \right)}{r_{L}+L+\left(r_{T}+r_{v}\right)\cos\left(\gamma \right)}$$

It is important to note that, when $r_{v}=0$, the posture stable control could still be achieved because the P point was still located above the total CoM due to the existence of leg mass.

#### 4.1.2. Forward Speed Control

**Figure 6:**
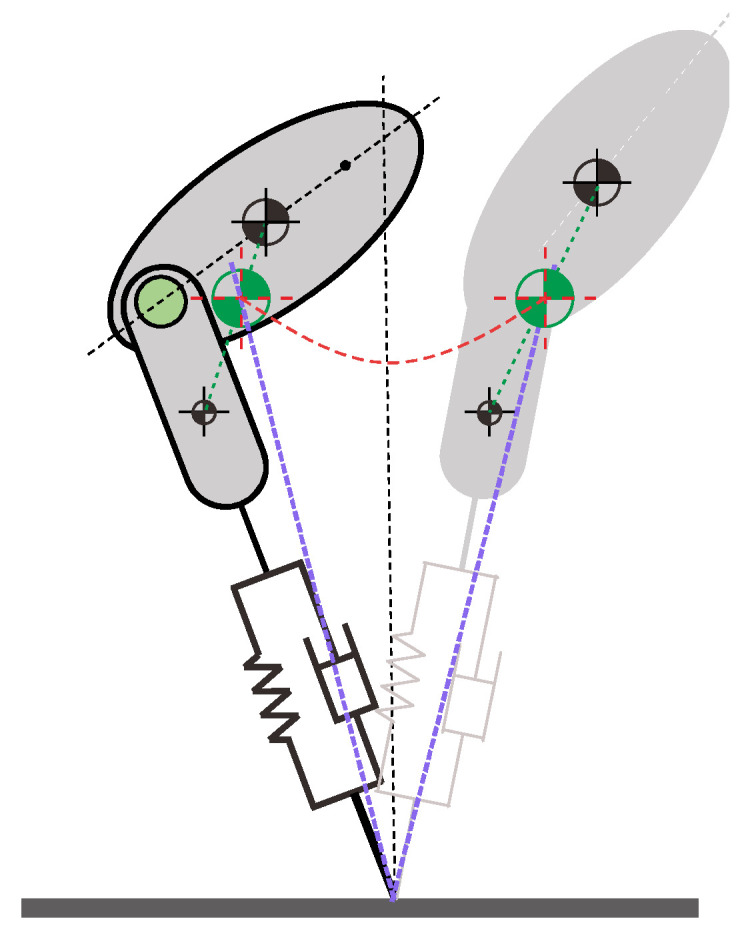
Sketch for asymmetry description.

As shown in Figure 6, the trajectory of the hip is not symmetric because the axis along the leg does not pass the total CoM, and few works could be found discussing the forward jumping or running strategy for the asymmetric extended SLIP model. In [22], a control method was proposed based on the concept of the virtual pendulum for the compliant bipedal robot, where the robot was modeled with a rigid torso and spring legs without mass, and limit cycles were searched with the angle of attack $\theta_{F}$ being fixed to a certain value. However, this method could not adaptively tune the angle of attack to autonomously track the desired forward speed. In [28,29], a velocity-based leg adjustment method (VBLA), which regulated the angle of attack adaptively according to the real-time feedback velocity of the CoM, was introduced for the walking and running of the SLIP model, as well as for robust hopping of the Trunk-SLIP (TSLIP) model [24]. However, in [24], stable forward jumping could not be achieved for the extended SLIP model because the desired forward speed was not considered in the control method. Raibert [14] presented a method to adjust the angle of attack for an asymmetric model where the leg mass was concentrated under the spring leg, which was different from the model in this work. To implement stable and accurate forward speed control of the compliant one-legged robot, we propose a foot placement method, which could adaptively regulate the angle of attack of the robot.

In [14], based on symmetry analysis, Raibert provided a foot placement method for the forward speed control of a one-legged robot model whose torso CoM coincides with the hip joint; however, it is inapplicable for the extended SLIP because the foot angles when the robot lifts off and touches down are not symmetrical anymore. Here, we improved the method by introducing an integral term to compensate for the forward speed tracking error introduced by the asymmetric characteristic of the extended SLIP model. The improved method was expressed as:

(13) $$x_{TD}\left(k+1\right)=k_{T}T_{st}\left(k\right){\dot{x}}_{T_{ap}}\left(k\right)+k_{v}\left({\dot{x}}_{T_{ap}}\left(k\right)-{\dot{x}}_{T_{des}}\right)\;\;+k_{I}\overset{k}{\underset{i=0}{\sum }}\left({\dot{x}}_{T_{ap}}\left(k\right)-{\dot{x}}_{T_{des}}\right)$$

(14) $$\theta_{Fdes}\left(k+1\right)=\arcsin\left(\frac{x_{TD}\left(k+1\right)}{L_{r}}\right)$$

where $x_{TD}\left(k+1\right),$ represents the point foot displacement, with respect to the hip joint in the *x*-direction, for the touchdown event of *k* + 1th jumping, $T_{st}\left(k\right),\;{\dot{x}}_{T_{ap}}\left(k\right)$ represent the stance period and the forward speed of the torso at the apex in *k*th jumping, and $k_{T},\;k_{v}$ represent the time coefficient and speed error coefficient; ${\dot{x}}_{T_{des}}$ represents the desired forward speed, the last term in the right hand of Equation (13) represents the integral of the tracking error of the forward speed, and $\theta_{Fdes}\left(k+1\right)$ represents the desired angle of attack for the next touchdown event.

To avoid disturbance due to inaccurate evaluation of the stance period $T_{st}$, it is updated using the last stance period $T_{st}\left(k\right)$. It is worth noting that the time coefficient $k_{T}$ did not need to be set to 1/2, which is different from Raibert’s method as well [14]. The introduced integral term could eliminate the steady-state tracking error and implement an accurate forward speed tracking performance.

Noting that the desired angle of attack could not be controlled directly, we controlled it indirectly through the servo control of the desired leg swing angle $\gamma_{des}$. The relationship between these two angles is $\gamma_{des}=-\theta_{T}+\theta_{Fdes}$, where$\;\theta_{T}$ could be measured by IMU. A PD control strategy is adopted to track the desired leg swing angle $\tau =k_{p}\left(\gamma -\gamma_{des}\right)+k_{d}\dot{\gamma }$, where$\;k_{p}$ and $k_{d}\;$are PD control parameters that need to be chosen carefully. Furthermore, it is worth mentioning that, due to the maximum output ability of the motor M, τ needs to be restricted to a certain range $\left[-\tau_{m},\tau_{m}\right].$

#### 4.1.3. Jumping Height Control

Publications could be found on the leg rest length regulation methods for tracking the hopping and running height of compliant legged robots. In [24], a deadbeat control strategy, which was based on the analytical solution of a simple vertical jumping model, was utilized for hopping height tracking control; however, it does not apply to the planar jumping described in this work. In [30], an energy-based leg rest length regulation method was proposed by Ioannis et al. for an energy-stabilized SLIP model, which is also different from the model described in this work. To make full use of the passive compliance of the spring-like leg and achieve accurate jumping height tracking for the extended SLIP model, an improved leg rest length regulation method, based on the one presented in [30], was introduced. 

We assume that γ has already been controlled to the desired position (i.e., there is no relative motion between the torso and leg) before the robot reaches the jumping apex, which is practically possible when the servo control for γ is good enough. Moreover, to maintain a stable posture in the flight phase, the angular velocity of the total robot should be sufficiently small. Under the above two conditions, it can be seen that the total rotation kinetic energy at the jumping apex is close to zero. Under the condition that the forward speed was accurately tracked to the desired value, the jumping height could be determined by the total system energy of the robot. Considering this, we adopted the energy-based leg rest length regulation law to control the jumping height in the stance phase:

(15) $$\{\begin{array}{c} {\dot{L}}_{r}=0,\dot{L}<0\\ {\dot{L}}_{r}=-k_{L_{r}}\dot{L}(E-E_{tar}),\dot{L}\geq 0 \end{array}$$

where $k_{L_{r}}$ represents the tuning coefficient of leg rest length, and *E* and $E_{tar}\;$ represent the feedback system energy and the target system energy for jumping height control, respectively. Leg rest length would be restricted to the range of $L_{rmin}<L_{r}<L_{rmax}$ due to the structure dimension limitation. The constraint condition $\dot{L}\geq 0$ was added to the regulation method, which is different from the method in [30], meaning that the leg rest length would be regulated only after the spring-like leg is compressed to the bottom in the stance phase, which makes the impact energy absorb sufficiently by the passive component of the robot and gradually injects or releases energy to track the desired total energy for jumping height control. 

In a certain limit cycle of the one-legged robot, the system energy *E*, in the flight phase, is constant when neglecting air resistance and joint damping. If one wants to track the desired jumping height, it is necessary to accurately determine the target system energy $E_{tar}$. However, not all states of a limit cycle of the robot with the proposed control strategy could be known previously as the states of the simple SLIP model, which means that the accurate target system energy could not be known previously. Thus, the method proposed in [30] would fail to accurately track the desired jumping height for the extended SLIP model. Here, an updating method of the target system energy was introduced, in which the target system energy was initiated by using the initial states of the robot, and iteratively updated based on jumping height error:

(16) $$E_{tar0}=m_{T}gH_{des}+m_{L}g\left(H_{des}-r_{T}-L_{1}\right)+\frac{1}{2}\left(m_{T}+m_{L}\right){\dot{x}}_{T_{des}}^{2}\;$$

(17) $$E_{tar}\left(k+1\right)=E_{tar}\left(k\right)+k_{E}\left(z_{Tap}\left(k\right)-H_{des}\right)$$

where $H_{des},\;{\dot{x}}_{T_{des}},E_{tar0}$ represent the desired jumping height, desired forward speed of the torso at the apex, and the estimated initial target system energy for a stable jumping; $E_{tar}\left(k\right),\;z_{T_{ap}}\left(k\right)$ represent the updated target system energy and feedback jumping height in the *k*th jumping; $k_{E}$ represents the energy updating coefficient based on jumping height error.

**Figure 7:**
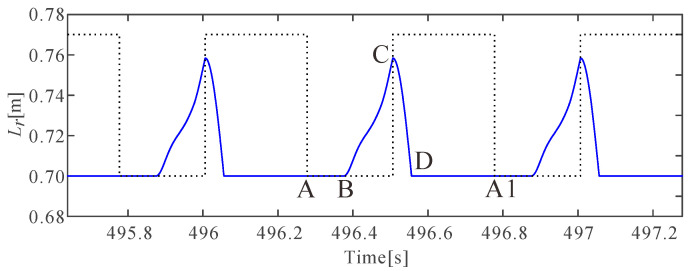
An example of the variation of leg rest length over time in stable periodical jumping.

The leg rest length could not be regulated using the dynamics control method in the flight phase because the foot mass was not taken into account. Therefore, a kinematic interpolation method was utilized to make the leg rest length retract to the initial value to prepare for the next touchdown event:

(18) $${\dot{L}}_{r}=(L_{r0}-L_{re})\frac{2\pi }{T_{m}}\sin(\frac{2\pi }{T_{m}}t),t\in [0,\frac{T_{m}}{4}]$$

where $L_{re}$ represents the end state of leg rest length in the stance phase; $T_{m}$ represents the period of the sine function, and it needs to be chosen properly to make sure the foot would not touch the ground during the leg swing process, as well as to make sure the leg rest length retracts to the initial value before touchdown. Figure 7 shows one of the leg rest length variations in stable jumping. The black dotted curve, which has two different values, was added to indicate the different phases in stable jumping, with the low value indicating the stance phase and the high value indicating the flight phase. The blue curve is a simulation result indicating the leg rest length variation over time. Specifically, from point A to A1, the blue curve indicates the leg rest length variations over time in a jumping period. From A to B, the leg rest length does not change, indicating the pure compression process of the passive compliant leg in the stance phase; from B to C, the leg stretches to inject energy for jumping height control in the stance phase; from C to D, the leg retracts after the legged robot lifts off and then waits for the next touchdown event.

To implement the stable and fast jumping of the robot, the desired forward speed and the jumping height, which were the control target values, were given first. We selected the jumping apex as the initial position of the extended SLIP model. By giving the initial states of the extended SLIP model properly, and tuning the control parameters well, stable and fast jumping could be achieved, and the given control target values could be tracked accurately.

### 4.2. Stability Analysis

The linearized Poincare map, which has been widely used to analyze the asymptotical stability of the limit cycles of legged robots [31,32], was adopted to analyze the stability of the jumping limit cycle of the legged robot. A brief introduction to using the linearized Poincare map for the jumping limit cycle in this work was described as follows. 

The Poincare surface of the jumping dynamics is selected as $S=\left\{x_{s}|\;x_{s}\in Q_{s},x_{s}\left(6\right)=\dot{L}=0\right\}$, namely the moment of maximum compression of the spring leg in the stance phase. Since the leg rest length was not changed during the compression phase, it does not need be taken into account. The Poincare map could be expressed as P:S→S, where $x_{s}\left(k+1\right)=P\left(x_{s}\left(k\right)\right)$. The fixed point about a limit cycle was expressed as $x_{s}^{*}=\left[\theta_{T}^{*};\gamma^{*};L^{*};{\dot{\theta }}_{T}^{*};{\dot{\gamma }}^{*};\;{\dot{L}}^{*}=0\;\right]$, which satisfied $x_{s}^{*}=P\left(x_{s}^{*}\right)$. The Poincare map has only five independent arguments since $\dot{L}=0$ is constant. 

The Poincare map could be linearized about the fixed point, with respect to the five independent arguments, as $x_{s}\left(k+1\right)-x_{s}^{*}=J_{P}\left(x_{s}\left(k\right)-x_{s}^{*}\right)$, where $J_{P}$ is the Jacobian of the Poincare map and could be computed as follows:

(19) $$J_{P}={\left[J_{P1},J_{P2},\ldots ,J_{P5}\right]}_{5\times 5}$$

(20) $$J_{Pi}=\frac{P\left(x^{*}+\delta x_{s,i}\right)-P\left(x^{*}+\delta x_{s,i}\right)}{2\delta x_{s,i}},\;i=1,2,\ldots ,5,$$

and $\delta x_{s,i}={\left[0;0;\ldots ;\delta x_{s,i};\ldots ;0\right]}_{5\times 1},\;i=1,2,\ldots ,5,\;\delta x_{s,i}>0$. The fixed point $x_{s}^{*}$ of the Poincare map is locally exponentially stable if and only if the eigenvalues of $J_{P}$ have magnitude strictly less than 1, i.e., ${\left|\lambda_{P}\right|}_{max}<1$.

## 5. Simulations and Results

This section aimed at validating the effectiveness of the proposed control strategy through numerical simulations. The jumping simulations of the compliant one-legged robot were conducted using MATLAB 2019b, and the dynamics functions were solved by the ODE45 solver. According to the real robot data, the restriction of hip torque and leg rest length was set as $\tau_{m}=60\;Nm$, $-{\dot{L}}_{\min}={\dot{L}}_{\max}=1\;m/s$, respectively. The model parameters of the extended SLIP model could be seen in Table 1. From Equations (12)–(18), control parameters need to be selected carefully to implement stable jumping, namely the stable posture control parameter $r_{v}$ in stance phase, the angle of attack control parameters $k_{T},\;k_{v},k_{I}$ and the hip servo control parameters $k_{p},k_{d}$ in flight phase, the jumping height control parameters $k_{L_{r}},k_{E}$, and the leg retraction coefficient $T_{m}$. To simplify the parameter tuning process, we fixed some of them as $r_{v}=0\;m,\;k_{v}=0.01{\;s}^{-1},\;\;k_{I}=0.01\;s^{-1},\;$ $k_{p}=$ $900\;\mathrm{Nm}\cdot {\mathrm{rad}}^{-1}$,$k_{d}=22\;\mathrm{Nm}\cdot {\mathrm{rad}}^{-1}\cdot s,\;k_{E}=-50\;J\cdot m^{-1},\;\;T_{m}=0.4\;s,\;T_{st0}=0.2\;s$, and then, there remain only two parameters $k_{T},\;k_{L_{r}}$ that need to be tuned. The simulation results shown below demonstrated the effectiveness of these settings.

To find the stable limit cycle of the robot with different control target values, the initial states of the robot were set as $x_{f}=\left(0;H_{des};0;0;{\dot{x}}_{T_{des}};0;0;0\right)$, which means the control target variables were initiated to the control target values, and other state variables were set to zero. Tuning the control parameters $k_{T},\;k_{L_{r}}$ well, the jumping of the legged robot would converge, to a certain limit cycle, from the initial states with a relatively fast convergence rate. An optimal method was utilized for auto-tuning the two control parameters described as follows:

(21) $$\begin{array}{c} min\\ \left(k_{T};\;k_{L}\right) \end{array}\;F=\overset{N=50}{\underset{k=1}{\sum }}\frac{k^{2}}{N^{2}}\times \left[{\left({\dot{x}}_{T_{ap}}\left(k\right)-v_{des}\right)}^{2}+{\left(z_{T_{ap}}\left(k\right)-H_{des}\right)}^{2}\right]$$

(22) $$s.\;t.\;\{\begin{array}{c} Given\;initial\;condition\\ Robot\;dynamics\;with\;the\;proposed\;controller \end{array}$$

where F represents the evaluation function value, and N represents the maximum preset jumping step. The evaluation function value is the weighted average of the tracking error at apexes, and the weight of the tracking error increases with the jumping steps; the weights are selected to make the jumping converge fast. If the robot falls before N step, a relatively large number, such as 100, would be added into *F* as punishment to make sure the robot converges to a stable jumping value. This optimal method was solved by the FMINCON function of MATLAB 2019b.

### 5.1. Control Precision and Stability

In this subsection, the desired jumping height and forward speed (i.e., control target values) were set as $H_{des}=1\;m,\;{\dot{x}}_{T_{des}}=1\;m\cdot s^{-1},$ and the initial states were set as $x_{f}=\left(0;1\;m;0;0;1\;m\cdot s^{-1};0;0;0\right)$, which means the one-legged robot was raised to 0.1 m above the ground and then allowed to fall freely with an initial forward speed of $1\;m\cdot s^{-1}$. The control parameters were optimized to obtain the minimum evaluation function value of Equation (21). 

**Figure 8:**
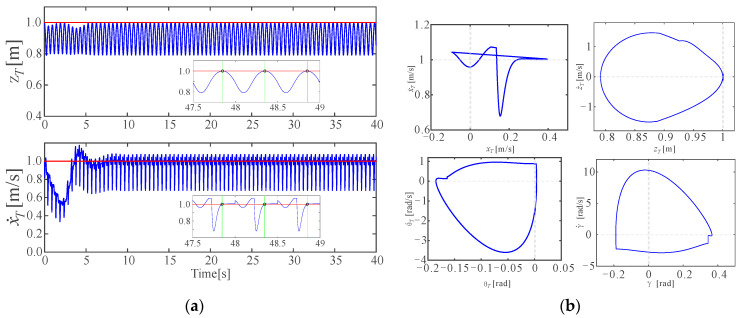
One of the stable and accurate control results: (**a**) The stable and accurate control target tracking results with steady states scaled up; (**b**) The phase graph of two state variables of the compliant one-legged robot in a stable jump, which could be seen to form a limit cycle.

Figure 8 presents the stable and accurate tracking results of the jumping height and the forward speed. In Figure 8a, the blue curves represent the real-time trajectories of the torso, the red horizontal lines represent the desired jumping height and desired forward speed, respectively, and the green vertical lines in the two enlarged subplots represent the moment, while the torso is arriving at the apexes, in which ${\dot{y}}_{T}=0$. It could be seen that the jumping height and the desired forward speed were tracked accurately at the apex after about 8 s, validating the accurateness of the proposed control strategy. Figure 8b shows the phase graph of four of the state variables, and it could be seen that a stable limit cycle was formed with the proposed control strategy.

The stability of the limit cycle above was discussed using the linearized Poincare map. The fixed point of the limit cycle on the Poincare surface was found as $x^{*}=\left[-0.1140;\;0.1448;\;0.3429;\;0.8498;-2.749\right]$, and the eigenvalue vector of the Jacobian matrix of the Poincare map was found as $\lambda_{P}=\left[0.354+0.853i;\;0.354+0.853i;-0.0036+0.125i;-0.0036+0.125i;0.488\right]$, where ${\left|\lambda_{P}\right|}_{max}=0.923<1$, meaning that the limit cycle is exponentially stable; thus, the jumping of the robot is also stable.

In order to show the advantages of the proposed control strategy for the extended SLIP model, two other comparative simulations with different control strategies were conducted. To make the posture stabilized, two comparative simulations were conducted using the VPP control method mentioned in Section 4.1.1. The differences between these control strategies are the control method of the forward speed and the jumping height, which were described, in detail, as follows.

Comparative simulation 1 (C1): For the control of the forward speed, Raibert’s method, based on symmetric analysis, without the integral term was adopted, and the initial estimated stance period $T_{st0}$ was not updated during the total simulation process; for the control of jumping height, the energy-based leg rest length regulation with constant estimated energy expressed in Equation (16) was adopted. This control strategy could be seen as a simple combination of the related method in [14,30]. The control parameters were tuned well to achieve stable jumping, which could be seen in Table 2. The initial conditions and remaining parameters were the same as the proposed strategy. 

Comparative simulation 2 (C2): The proposed forward speed control method with the integral term was adopted for the forward speed control, and the energy-based leg rest length regulation, with constant estimated energy expressed in Equation (16), was adopted for the jumping height control. This control strategy was set to compare the effectiveness of the energy updating method for the accurate control of jumping height. The control parameters were tuned well to achieve stable jumping, which could be seen in Table 2. The remaining parameters were the same as the proposed strategy.

**Figure 9:**
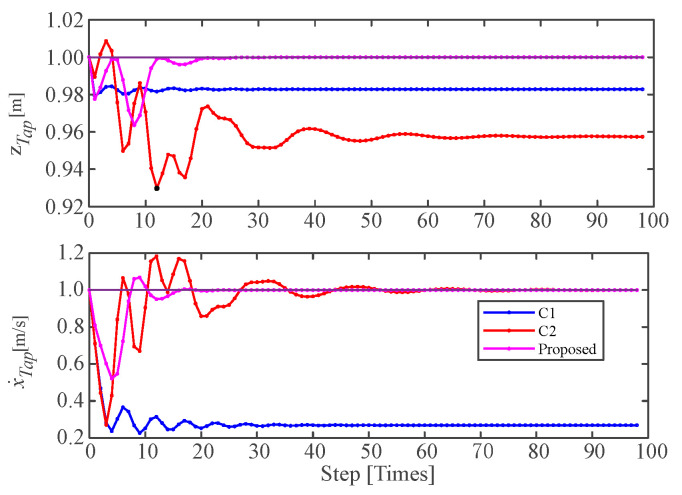
Comparative simulations with different control strategies.

Figure 9 depicted the variations of the control target variables, i.e., jumping height and forward speed at the apex, with different control strategies. The three control strategies could lead to a stable jumping for the extended SLIP model; however, the control accuracy was different from each other. The result of C1 indicates that neither the desired jumping height, nor the desired forward speed, could be tracked accurately, which means that the simple combination of the existing methods in [14,30] could not achieve the desired jumping for the extended SLIP model. Comparing C2 to C1, the desired forward speed could be tracked accurately by introducing the integral term of the jumping speed, though the jumping height could not be tracked accurately, which is due to the inaccurately estimated system energy in a stable limit cycle. Comparing the proposed method to C2, by introducing the system energy updating method, both the desired forward speed and the desired jumping height could be tracked accurately, demonstrating the effectiveness of the proposed control strategy for accurate jumping height and forward speed tracking for the extended SLIP model.

### 5.2. Fast and Robust Jumping

Generally, we care about the jumping speed of the robot more than the jumping height. To further test the ability of the one-legged robot with the proposed control strategy toward fast jumping, different forward speed tracking simulations were conducted. In the following simulations, the forward speed was selected as the main control target, and the desired jumping height was fixed as $H_{des}$= 1 m. 

Table 3 shows the optimal jumping results with different desired forward speeds. The optimal time coefficient $k_{T}$ needs not to be 0.5, which is considered to be due to the asymmetric properties of the extended SLIP model, and it played a role in the optimization of the jumping performance. With the proposed controller, and without the restriction of the ground fraction cone, the robot could be seen implementing different forward speeds, from zero to a fast speed of 1.75 m/s (6.3 km/h), which demonstrated the good performance of the proposed controller for fast stable jumping of the extended SLIP model. On the other hand, it could be seen that the control parameters, evaluation function values *F*, and the eigenvalues $\lambda_{P}$ of the Jacobian matrixes do not exhibit strong regularity with respect to desired forward speeds, which was considered to be due to the similar but different initial conditions.

The robustness of stable limit cycles with different forward speeds was explored as well. In this work, the robustness was indicated by the admitted single-state perturbation ranges of the state variables in limit cycles, within which the jumping would still converge to the limit cycle. The initial conditions of the legged robot were set as the apex states of the limit cycles, with different desired forward speeds in previous simulations, but the control parameters remained the same as those in the related limit cycles. It is worth noting that the event-based variables and the integral term in Equations (13) and (17) should be initiated by those in related limit cycles as well because they also influence the locomotion of the robot. Then, the perturbation would be added to one of the state variables at the apex of the robot in a single simulation, and we found the maximum and minimum admitted perturbations in which the robot could recover to the stable jumping. To simplify the simulation process, the four typical state variables $\left(z_{T};\theta_{T};{\dot{x}}_{T};{\dot{\theta }}_{T}\right)$ would be selected to be tested. The perturbations of $x_{T}$ would not influent the stability of the jumping, and the perturbations of $\left(\gamma ;\dot{\gamma }\right)$ could be transferred to those of $\left(\theta_{T};{\dot{\theta }}_{T}\right)$ because the target angle of attack is always constant during the flight down phase.

**Figure 10:**
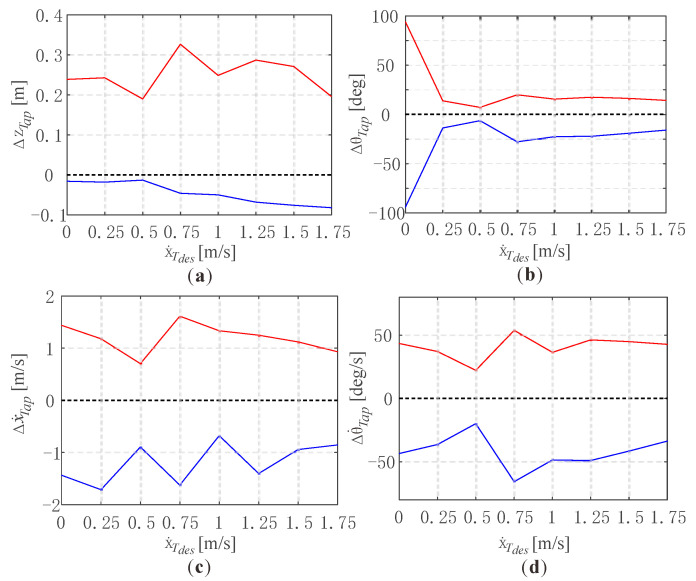
Admitted single-state perturbation ranges of four different state variables of the torso at the apex, with respect to different desired forward speeds: (**a**) Admitted perturbation ranges of the jumping height; (**b**) Admitted perturbation ranges of the orientation of the torso; (**c**) Admitted perturbation ranges of the forward speed of the torso; (**d**) Admitted perturbation ranges of the angular velocity of the torso.

Figure 10 shows the admitted single-state perturbation ranges of the four different state variables, with respect to different desired forward speeds. The black dotted lines represent the zero perturbations of the related stable limit cycle with different desired forward speeds, and the red and blue curves represent the upper and the lower boundary of the admitted perturbations in which the robot could remain stable jumping. It could be seen that the robot could recover to a stable jumping state with relatively large single-state perturbations. Figure 10a depicted the admitted single-state perturbation ranges of the jumping height $z_{T_{ap}}$. It could be seen that the robot could withstand large positive perturbations of jumping height almost above two times the net jumping height, i.e., 0.1 m. However, the admitted negative perturbation ranges are relatively low and increase slightly as the desired forward speed. Figure 10b depicted that the robot could withstand about 20-degree positive perturbations of the torso orientation at the apex. Specifically, it is remarkable that the admitted perturbation of the limit cycle with the 0 m/s desired forward speed is about ±90°, which is considered to inherit the advantages of the VPP controller for the vertical hopping presented in [24]. Figure 10c,d depicted the admitted single-state perturbation ranges of the forward speed and the torso angular velocity, which are nearly 1 m/s and 50 deg/s, and both are relatively large. The analysis above validates the robustness of the stable jumping.

**Figure 11:**
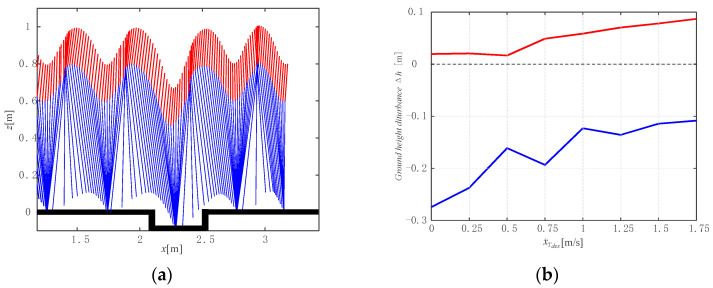
The robustness of jumping on uneven terrain: (**a**) A snapshot of the robot jumping on one-step uneven terrain; (**b**) The admitted ranges of ground height disturbances where jumping remains stable, with respect to different desired forward speeds.

To further test the robustness, we tested the robot jumping on uneven terrain with a one-step disturbance of ground height, as shown in Figure 11a. The initial conditions of the legged robot were set as apex states of the limit cycle with different desired forward speeds in previous simulations. In this part, we assumed that the foot would not collide with the edge of the uneven terrain, the jumping height of the torso was set as 1 m, which means the net jump height of the torso is 0.1 m. Figure 11a shows the snapshot of jumping on uneven terrain with one-step ground height disturbance Δh. 

Figure 11b shows the admitted disturbance ranges of the ground height with respect to different desired forward speeds within which the robot maintains stable jumping. The red curve denotes the maximum admitted positive disturbances of ground height, and the blue curve denotes the minimum admitted negative disturbances of ground height. The stable jumping could be maintained with a certain range of ground height disturbance, where the higher the forward speed, the larger the upper boundary of the admittance disturbance and the smaller the lower boundary of the admittance disturbance. The stable jumping could withstand the negative ground height disturbance above 100% of the net jumping height of the torso (0.1 m), which is larger than the positive one. The simulation result demonstrates the robustness of the robot jumping on uneven terrain. 

## 6. Conclusions and Future Work

This work proposed an improved control strategy for the stable and fast planar jumping of a designed compliant one-legged robot. The robot was modeled as an extended spring-loaded inverted pendulum (SLIP) with non-negligible torso inertia, leg inertia, and leg damping. The posture stable control was achieved by introducing the approximate VPPC-FP method. An improved foot placement method with variable time coefficient and integral term of the forward speed tracking error was introduced to accurately track the forward speed. A modified energy-based leg rest length regulation law was adopted, in which the integral term of jumping tracking error was also introduced to accurately track the jumping height. A practical stability criterion was introduced for the judgment of the jumping stability. 

Numerical simulations were conducted to validate the effectiveness of the proposed control strategy. Results show that stable and fast planar jumping of the compliant one-legged robot could be implemented based on the extended SLIP model and the proposed control strategy. The jumping height and forward speed of the torso at the apex could be tracked accurately. The robot could recover to stable jumping from certain disturbances of state variables or uneven terrains.

In the next step, experiments will be conducted on our compliant one-legged robot for validating the effectiveness of the proposed control strategy in practice. In the long term, we will build a biped robot with passive compliance and extend the control strategy to a stable and fast bipedal robot’s jumping and running.

## Figures and Tables

**Table 1:** Design parameters of our compliant one-legged robot.

Term	Symbol	Value [Units]
Torso mass	$m_{T}$	12 [kg]
Torso’s moment of inertia	$I_{T}$	1.0 [kg·m^2^]
Distance from hip to torso CoM	$r_{T}$	0.2 [m]
Leg mass	$m_{L}$	6.5 [kg]
Leg’s moment of inertia	$I_{L}$	0.188 [kg·m^2^]
Distance from hip to leg CoM	$r_{L}$	0.25 [m]
Initial leg rest length	$r_{L}+L_{r0}$	0.7 [m]
Leg stiffness	k	6000 [N/m]
Leg damping	c	60 [N/ (m·s^−1^)]

**Table 2:** The value of the control parameters of different control strategies.

Method	$k_{T}$	$k_{L}$	$k_{v}$	$k_{I}$	$k_{E}$
C1	0.5	0.04	0.003	0	0
C2	0.5	0.1	0.015	0.01	0
Proposed	0.45	0.062	0.01	0.01	−50

**Table 3:** The optimal control parameters and results with different desired forward speeds.

Item	0.00 m/s ^1^	0.25 m/s	0.50 m/s	0.75 m/s	1.00 m/s	1.25 m/s	1.50 m/s	1.75 m/s
$k_{T}$	0.3600	0.3636	0.3769	0.4200	0.4533	0.4368	0.4237	0.4400
$k_{L}$	0.1000	0.1023	0.1189	0.0320	0.0620	0.0221	0.0196	0.0150
F	—	0.0535	0.0628	0.0555	0.0483	0.0612	0.1711	4.0591
$\lambda_{P}$	0.978	0.963	0.953	0.983	0.923	0.907	0.852	0.872

^1^ Jumping with the desired speed of 0 m/s and the initial conditions above, $k_{T}$ and $k_{L}$ could be set as attributable values, to make it stable with disturbance, we used the parameters as those of the stable jumping with the desired forward speed of 0.25 m/s.

## Data Availability

Not applicable.
